# Comprehensive analysis of mortality risk factors in low-grade B-cell lymphoma

**DOI:** 10.1371/journal.pone.0328666

**Published:** 2026-03-04

**Authors:** Tong-Yoon Kim, Gi-June Min, Seok-Goo Cho, Seoree Kim, Jong Hyuk Lee, Byung-Su Kim, Joon Won Jeoung, Hye Sung Won, Youngwoo Jeon

**Affiliations:** 1 Department Of Hematology, Yeoido St. Mary’s Hospital, College of Medicine, The Catholic University of Korea, Seoul, Republic of Korea; 2 Department Of Hematology, Seoul St. Mary’s Hospital, College of Medicine, The Catholic University of Korea, Seoul, Republic of Korea; 3 Department Of Oncology, Bucheon St. Mary’s Hospital, College of Medicine, The Catholic University of Korea, Seoul, Republic of Korea; 4 Department Of Hematology, Incheon St. Mary’s Hospital, College of Medicine, The Catholic University of Korea, Seoul, Republic of Korea; 5 Department Of Hematology, Eunpyeong St. Mary’s Hospital, College of Medicine, The Catholic University of Korea, Seoul, Republic of Korea; 6 Department Of Oncology, Daejeon St. Mary’s Hospital, College of Medicine, The Catholic University of Korea, Seoul, Republic of Korea; 7 Department Of Oncology, Uijeongbu St. Mary’s Hospital, College of Medicine, The Catholic University of Korea, Seoul, Republic of Korea; Azienda Ospedaliera Universitaria SS Antonio e Biagio e Cesare Arrigo, Alessandria, University of Eastern Pedemont, ITALY

## Abstract

**Background:**

Low-grade B-cell lymphomas (LGBCLs) account for approximately 40% of non-Hodgkin lymphomas with low progression. LGBCL is divided into subgroups, which share common complications. Analyzing prognostic factors and mortality causes could improve patient survival; however, currently available models present limitations in discriminating the cause of death. Therefore, this study aimed to compare the prognostic factors and causes of death, such as secondary malignancies (SMs), aggressive histologic transformation (HT), and infectious complications, including coronavirus disease 2019 (COVID-19), in LGBCLs using a competing risk analysis.

**Methods:**

This retrospective analysis included 1,047 adults with LGBCLs (follicular lymphoma, 689; marginal zone lymphoma, 312; mantle cell lymphoma [MCL], 46) diagnosed between January 2011 and December 2022 across seven centers. Competing risk models were employed to estimate cumulative incidence rates of lymphoma progression-related and non-lymphoma-related mortality.

**Results:**

Patients with SMs (3.8%) exhibited poorer overall survival than those without SMs, whereas HT and COVID-19 status did not impact survival outcomes in multivariate analysis. Analysis revealed an association of SMs, age > 60 years, male sex, pleural effusion, and elevated lactate dehydrogenase levels with worse non-lymphoma-related mortality. Moreover, age > 60 years, MCL, nodal MZL, and anemia were linked to poorer outcomes for lymphoma progression-related death.

**Conclusions:**

The management of non-lymphoma-related risk factors, such as through early SM detection, is crucial for improving the survival of patients with LGBCLs.

## Introduction

Low-grade B-cell lymphomas (LGBCLs) represent a heterogeneous group of indolent non-Hodgkin lymphomas, accounting for approximately 40% of all non-Hodgkin lymphomas globally. These malignancies are characterized by their indolent course and prolonged survival rates [1]. However, the epidemiological landscape of LGBCL subtypes exhibits regional variations. The incidence of follicular lymphoma (FL) and mantle cell lymphoma (MCL) in Northeast Asia has been increasing, underscoring the dynamic nature of LGBCL epidemiology [2–4]. The advent of anti-CD20 monoclonal antibodies in the late 1990s revolutionized LGBCL treatment, substantially enhancing patients’ progression-free survival [5,6]. However, this advancement has shed light on a critical gap in our understanding of LGBCL outcomes. While lymphoma progression can be effectively suppressed, overall survival (OS) improvements are yet to be proportionate. This discrepancy is primarily attributed to non-lymphoma-related mortality (NLM), which has emerged as a major impediment to improving long-term outcomes in patients with LGBCLs [7–9].

Current prognostic models for LGBCLs, including the Follicular Lymphoma International Prognostic Index (FLIPI) [10], the mucosa-associated lymphoid tissue (MALT) lymphoma prognostic index (MALT-IPI) [11], and the mantle cell lymphoma International Prognostic Index (MIPI) [12], have been valuable in predicting survival. However, these models have inherent limitations; notably, they fail to distinguish between lymphoma-related mortality and NLM, potentially obscuring the actual effects of various risk factors on different causes of death.

To address this critical knowledge gap, a more nuanced analysis of prognostic factors based on specific causes of death in patients with LGBCLs is crucial. Key contributors to NLM share common complications across histologic subtypes, including secondary malignancies (SMs) [13,14], histologic transformation (HT) to aggressive lymphoma, and infectious complications. The latter has gained renewed importance considering the recent global coronavirus disease 2019 (COVID-19) pandemic. COVID-19 has introduced unprecedented challenges in managing patients with LGBCLs, potentially contributing to increased NLM rates. This evolving landscape of infectious risks, exemplified but not limited to COVID-19, underscores the necessity for a comprehensive reassessment of mortality factors in LGBCLs [15]. Furthermore, the complex interplay between various causes of death in LGBCLs necessitates advanced analytical approaches. Traditional statistical methods are limited in their ability to capture the nuanced relationships between risk factors and outcomes in the presence of competing risks. Consequently, in this study, we employed competing risk analysis and machine-learning algorithms to provide deeper insights into these interrelated mortality events [16,17].

The primary objective of this study was to evaluate the independent effects of prognostic variables and specific causes of death on survival outcomes in patients with LGBCLs. We aimed to identify key factors contributing to NLM, focusing on SMs, histologic transformation, and COVID-19-related infection. We sought to develop a more accurate prognostic model, distinguishing between lymphoma-related mortality and NLM. By providing a nuanced understanding of mortality risk factors in the context of both established and emerging health challenges, we strive to inform personalized treatment strategies and improve patient outcomes in LGBCL.

## Materials and methods

### Patient selection

In this multicenter retrospective study, we analyzed data of 1,047 patients with LGBCLs diagnosed and admitted between January 2011 and December 2022 across seven hospitals in the Republic of Korea (Yeoido, Seoul, Bucheon, Incheon, Daejeon, Eunpyeong, and Uijeongbu St. Mary’s Hospitals); the follow-up period ended on 31 May 2024. The medical records of all patients were accessed and extracted for research purposes between 1 June 2024 and 27 June 2024. The authors had access to information that did not identify individual participants during or after data collection. The following inclusion criteria were applied: (1) patients aged ≥ 18 years; (2) those diagnosed with FL (grade 1, 2, or 3A), splenic marginal zone lymphoma (MZL), extranodal MZL of mucosa-associated lymphoid tissue type, nodal MZL, or MCL; and (3) those who completed major clinical laboratory or radiology workup at the hospital. Essential evaluations included the complete blood count, serum lactate dehydrogenase (LDH) level, bone marrow biopsy, and computed tomography scans of the neck, chest, abdomen, and pelvis. Patients who had chronic lymphocytic leukemia, small lymphocytic lymphoma, FL grade 3B, or lymphoplasmacytic lymphoma were excluded. LGBCL treatment strategies comprised the watch-and-wait strategy, radiotherapy, or chemotherapy. First-line chemotherapy included RCVP (rituximab, cyclophosphamide, vincristine, and prednisone), R-CHOP (rituximab, cyclophosphamide, doxorubicin, vincristine, and prednisone), and BR (bendamustine and rituximab). A detailed CONSORT diagram is presented in S1 Fig.

This study was approved by the Institutional Review Board and Ethics Committee of the Catholic Medical Center in South Korea (approval no: SC23WISI0093; approval date: June 28, 2023). The requirement for patient informed consent was waived by the review board because of the retrospective nature of the study. All methods were performed in accordance with relevant guidelines and regulations.

Demographic, clinical, and pathologic data were collected through a comprehensive review of patients’ medical records. Variables were collected based on established prognostic indices, including the FLIPI, MALT-IPI, MIPI, and Groupe d’Etude des Lymphomes Folliculaires Criteria [18]. The collected variables included demographics (including sex and age at diagnosis), clinical traits (including the number of nodal sites, Ann Arbor Stage, pathological type, bone marrow involvement, and pleural effusion), and laboratory findings (including LDH and hemoglobin levels). Staging was assessed using the Lugano classification, which provides a standardized approach for initial evaluation, staging, and response assessment in patients with lymphoma. The treatment response was evaluated using the Deauville score, a 5-point scale, to assess the metabolic response on fludeoxyglucose-18 positron emission tomography. These standardized methods ensured consistency in patient evaluation across all participating centers [19,20].

### Statistical analysis

Categorical variables regarding patient characteristics were analyzed using the chi-squared or Fisher’s exact test, and continuous variables were compared between groups using Student’s *t*-test. Survival outcomes were evaluated based on OS. According to the Kaplan–Meier survival curve, OS was calculated as the proportion of patients who survived at the end of the follow-up period. Cox proportional hazard models were used in univariate and multivariate analyses to compare the risk factors affecting OS. The cumulative incidence of death was analyzed for progression-related death (CIP) and NLM. CIP and NLM were estimated based on the cumulative incidence of competing events using the Gray test for univariate analysis and Fine–Gray proportional hazard regression for multivariate analysis. Factors that significantly affected mortality in the univariate analysis (p < 0.05) were used in the multivariate analysis to determine their combined effect. Aalen’s additive regression model estimated the time-course effect of these factors [21].

To characterize the evolution of covariate effects over follow-up, we fitted Aalen’s additive hazards models on the event-time grid and derived the instantaneous absolute risk difference curves β(t) for each covariate. The cumulative coefficients B(t) from model were converted to β(t) by ΔB/Δt and smoothed with LOESS (span 0.35) to reduce jaggedness. We summarized each β(t) curve by (i) t_peak (time of maximum smoothed effect), (ii) t_zero-cross (first time the smoothed effect crossed from positive to ≤0), and (iii) a plateau interval [t_plateau−start,t_plateau−end], defined as the longest continuous segment after t_peak in which the centred 7-point moving average of ∣β(t)∣ remained below a data-adaptive threshold (with α = 0.01 and ε = 0.03):

$\text{max}\left\{z_{\text{1}-\alpha /\text{2}}\times \text{Median Absolute Deviation}(\text{raw }\beta -\text{smoothed }\beta ),\;\;\varepsilon_{\text{frac}}\times \text{max}|\beta (t)|\right\}$ For visual comparison by disease subtype, we additionally plotted overlay panels (FL, MZL, MCL) for four clinical covariates (second malignancy [SM], age ≥ 60 y, LDH elevation, anemia [Hb < 12 g/dL]) using the same modelling and smoothing settings. Y‑axis scales differ by panel.

We employed a random forest survival model in the competing risk setting to further explore the complex interplay between risk factors and outcomes. This machine-learning approach can capture non-linear relationships and interactions between variables [22]. Harrel’s concordance index (c-index) was calculated to assess the discriminative power of the model [23]. C-indexes close to 0.5 indicate random assumption and c-indexes of 1 represent perfect estimation. Two-sided p-values < 0.05 indicated statistical significance. All statistical analyses were performed using R version 4.0.2 (R Foundation for Statistical Computing, 2017).

## Results

### Patient characteristics

Table 1 summarizes the characteristics of the 1,047 patients. The median age at diagnosis was 53 (range: 18–94) years. The histological subtypes were distributed as follows: FL (n = 689, 65.8%), MZL (n = 312, 29.8%), and MCL (n = 46, 4.4%). We further analyzed three subgroups: patients with aggressive HT (n = 27, 2.6%), patients with SM (n = 40, 3.8%), and remaining patients (control; n = 980, 93.6%).

**Table 1:** Patient characteristics.

	Total	SM (n = 40)	HT (n = 27)	Control (n = 980)	SM vs. control, p	HT vs. control, p	SM vs. HT, p
**Diagnosis subtype, N (%)**					0.181	0.006	0.113
**FL**	689 (65.8)	21 (52.5)	15 (55.6)	653 (66.6)			
**Extranodal MZL**	206 (19.7)	10 (25.0)	4 (14.8)	192 (19.6)			
**Nodal MZL**	106 (10.1)	5 (12.5)	8 (29.6)	93 (9.5)			
**MCL**	46 (4.4)	4 (10.0)	0 (0.0)	42 (4.3)			
**First-line treatment, n (%)**					0.003	0.055	0.018
**BR**	381 (36.4)	8 (20.0)	6 (22.2)	367 (37.4)			
**R-CHOP**	154 (14.7)	2 (5.0)	6 (22.2)	146 (14.9)			
**RCVP**	209 (20.0)	9 (22.5)	10 (37.0)	190 (19.4)			
**RTx or W&W**	303 (28.9)	21 (52.5)	5 (18.5)	277 (28.3)			
**COVID-19, n (%)**	213 (20.3)	6 (15.0)	4 (14.8)	203 (20.7)	0.498	0.612	>0.999
**Age > 60 years, n (%)**	312 (29.8)	23 (57.5)	11 (40.7)	278 (28.4)	<0.001	0.235	0.273
**Female, n (%)**	536 (51.2)	20 (50.0)	12 (44.4)	504 (51.4)	0.987	0.602	0.844
**Involvement >4 nodal sites, n (%)**	271 (25.9)	6 (15.0)	9 (33.3)	256 (26.1)	0.163	0.537	0.142
**Bone marrow** **involvement, n (%)**	339 (32.4)	12 (30.0)	12 (44.4)	315 (32.1)	0.911	0.255	0.342
**Spleen involvement, n (%)**	165 (15.8)	3 (7.5)	6 (22.2)	156 (15.9)	0.224	0.539	0.171
**Pleural involvement, n (%)**	37 (3.5)	3 (7.5)	4 (14.8)	30 (3.1)	0.272	0.005	0.58
**LDH elevation, n (%)**	190 (18.1)	9 (22.5)	10 (37.0)	171 (17.4)	0.542	0.018	0.308
**Hemoglobin <12 g/dL, n (%)**	143 (13.7)	7 (17.5)	11 (40.7)	125 (12.8)	0.525	<0.001	0.068
**Ann Arbor Stage III–IV, n (%)**	620 (59.2)	18 (45.0)	20 (74.1)	582 (59.4)	0.099	0.181	0.035

BR, bendamustine and rituximab; FL, follicular lymphoma; HT, aggressive histologic transformation; LDH, lactate dehydrogenase; MCL, mantle cell lymphoma; MZL, marginal zone lymphoma; N, number; p, p-value; R-CHOP, rituximab, cyclophosphamide, doxorubicin, vincristine, and prednisone; RCVP, rituximab, cyclophosphamide, vincristine, and prednisolone; RTx, radiotherapy; SM, secondary malignancy group; W&W, watch and wait.

Compared with the other groups, the SM group had a significantly higher proportion of patients aged over 61 years (p < 0.001). The HT group showed a significantly higher prevalence of several adverse prognostic factors, including pleural effusion (p = 0.005), elevated LDH level (p = 0.018), anemia (p < 0.001), and advanced-stage disease (p = 0.035), compared with the SM group.

### Survival outcomes and risk factors in patients

**Fig 1:**
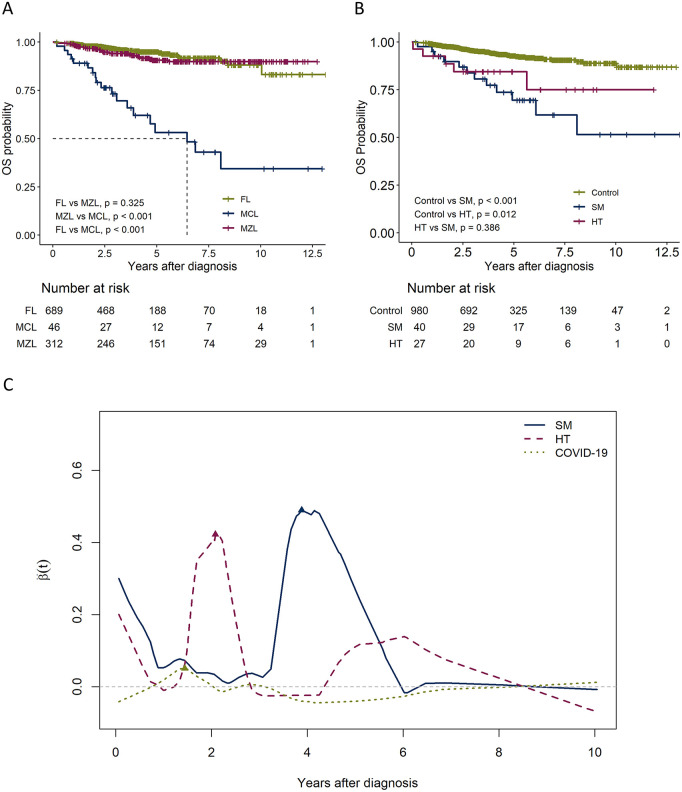
Survival outcomes for patients with low-grade B-cell lymphoma. **(a)** Overall survival (OS) by subtypes. **(b)** OS for the SM, HT, and other groups. **(c)** time‑varying additive‑hazards effects of time‑updated events for the SM, HT, and COVID19 groups. FL, follicular lymphoma; MZL, marginal zone lymphoma; MCL, mantle cell lymphoma; SM, secondary malignancy group; HT, aggressive histologic transformation group.

The median follow-up period was 4 (1–13) years. The 4-year OS rates were 95.4% (95% confidence interval [CI]: 93.6%–97.3%), 93.1% (95% CI: 90.1%–96.2%), and 62.0% (95% CI: 47.5%–80.9%) for FL, MZL, and MCL, respectively (Fig 1A). In the subgroup analysis, the 4-year OS rates were 77.9% (95% CI: 65.4%–92.9%) and 87.4% (95% CI: 71.3%–99.8%) for the SM and HT groups, respectively (Fig 1B). In the time‑varying additive‑hazards overlay, the instantaneous absolute risk difference β(t) for the second malignancy SM showed a prominent mid‑term rise, peaking around years 3–5 (β ≈ 0.45–0.50). Histologic transformation (HT) displayed an earlier peak centered near ~2 years. Late oscillations toward 8–10 years were interpreted cautiously, given the shrinking risk sets. The COVID‑19 curve remained low‑amplitude and near zero throughout, with a minor early blip (~1 year) and no sustained signal thereafter (Fig 1C).

The type of first-line chemotherapy regimen did not significantly affect OS (p = 0.234, S2 Fig in S1 File). COVID-19 did not significantly affect OS in the total patient cohort (p = 0.934); however, it was associated with poorer outcomes in patients who survived until January 2021 (p = 0.003, S2 Fig in S1 File).

Among 77 observed mortalities, 33 (42.9%) were due to lymphoma progression, 17 (22.1%) to infection, 13 (16.9%) to COVID-19 pneumonia, and 14 (18.1%) to other causes. The majority of non-relapse mortality occurred in 2022 (S3 Fig in S1 File). In the SM group, hepatobiliary and colorectal cancers were associated with poor outcomes (S3 Fig in S1 File).

In the multivariate Cox regression analysis, factors significantly associated with poorer OS included the presence of SMs (hazard ratio [HR]: 2.61, 95% CI: 1.15–5.92, p = 0.021), age over 60 years (HR: 3.52, 95% CI: 2.11–5.89, p < 0.001), male sex (HR: 1.67, 95% CI: 1.01–2.78, p = 0.047), MCL subtype (HR: 3.08, 95% CI: 1.58–6.00, p < 0.001), nodal MZL subtype (HR: 2.43, 95% CI: 1.27–4.65, p = 0.007), lymph node > 4 sites (HR: 2.2, 95% CI: 1.21–4.00, p = 0.010), pleural effusion (HR: 3.87, 95% CI: 1.93–7.76, p < 0.001), elevated LDH level (HR: 2.6, 95% CI: 1.58–4.29, p < 0.001), and anemia (HR: 2.02, 95% CI: 1.19–3.45, p = 0.010) (Table 2).

**Table 2:** Univariate and multivariate analyses of survival outcomes.

	Univariate		Multivariate	
	HR (95% CI)	p	HR (95% CI)	p
**Overall survival**				
**SM group vs. other**	4.23 (2.11–8.49)	<0.001	2.61 (1.15–5.92)	0.021
**HT group vs. other**	2.62 (1.06–6.48)	0.037	0.82 (0.29–2.29)	0.701
**Age > 60 years vs. ≤ 60 years**	4.36 (2.75–6.92)	<0.001	3.52 (2.11–5.89)	<0.001
**Male vs. female**	2.20 (1.36–3.54)	0.001	1.67 (1.01–2.78)	0.047
**MCL vs. other subtypes**	8.13 (4.84–13.7)	<0.001	3.08 (1.58–6.00)	<0.001
**Nodal MZL vs. other subtypes**	2.39 (1.36–4.21)	0.002	2.43 (1.27–4.65)	0.007
**Lymph node >4 sites vs. 4 sites**	2.96 (1.89–4.65)	<0.001	2.2 (1.21–4.00)	0.010
**BM involvement vs. none**	2.57 (1.63–4.03)	<0.001	1.04 (0.60–1.80)	0.880
**Splenomegaly vs. other**	2.51 (1.50–4.19)	<0.001	1 (0.55–1.85)	>0.999
**Pleural effusion vs. other**	4.95 (2.62–9.38)	<0.001	3.87 (1.93–7.76)	<0.001
**LDH elevation vs. none**	5.17 (3.30–8.09)	<0.001	2.6 (1.58–4.29)	<0.001
**Hemoglobin <12 g/dL vs. ≥ 12 g/dL**	5.03 (3.18–7.97)	<0.001	2.02 (1.19–3.45)	0.010
**Ann Arbor Stage III–IV vs. I–II**	2.91 (1.71–4.95)	<0.001	1.04 (0.51–2.10)	>0.999
**COVID-19 vs. none**	0.97 (0.52–1.81)	0.934		
**Non-lymphoma-related mortality**				
**SM group vs. other**	1.79 (0.91–3.54)	<0.001	4.81 (2.16–10.72)	<0.001
**HT group vs. other**	2.72 (0.79–9.32)	0.110		
**Age > 60 years vs. ≤ 60 years**	4.41 (2.40–8.09)	<0.001	3.39 (1.64–7.00)	0.001
**Male vs. female**	2.25 (1.19–4.25)	0.012	1.99 (1.03–3.84)	0.040
**MCL vs. other subtypes**	3.93 (1.76–8.76)	<0.001	1.37 (0.59–3.20)	0.460
**Nodal MZL vs. other subtypes**	2.15 (0.99–4.64)	0.051		
**Lymph node >4 sites vs. 4 sites**	2.24 (1.22–4.12)	0.009	1.62 (0.74–3.59)	0.230
**BM involvement vs. none**	2.10 (1.15–3.84)	0.015	1.26 (0.62–2.54)	0.520
**Splenomegaly vs. other**	1.05 (0.44–2.48)	0.910		
**Pleura effusion vs. other**	6.35 (3.02–13.33)	<0.001	5.45 (2.29–12.95)	<0.001
**LDH elevation vs. none**	3.93 (2.18–7.10)	<0.001	2.38 (1.25–4.53)	0.009
**Hemoglobin <12 g/dL vs. ≥ 12 g/dL**	3.00 (1.57–5.74)	0.001	1.17 (0.57–2.41)	0.670
**Ann Arbor Stage III–IV vs. I–II**	1.65 (0.87–3.12)	0.120		
**COVID-19 vs. none**	1.79 (0.91–3.54)	0.094		
**Cumulative incidence of lymphoma progression mortality**				
**SM group vs. other**	0.16 (0.02–1.17)	0.680		
**HT group vs. other**	2.25 (0.55–9.13)	0.260		
**Age > 60 years vs. ≤ 60 years**	3.86 (1.91–7.79)	<0.001	2.32 (1.04–5.16)	0.039
**Male vs. female**	2.06 (1.00–4.24)	0.050		
**MCL vs. other subtypes**	13.2 (6.53–26.68)	<0.001	5.93 (2.58–13.65)	<0.001
**Nodal MZL vs. other subtypes**	2.54 (1.11–5.78)	0.027	2.64 (0.97–7.19)	0.058
**Lymph node >4 sites vs. 4 sites**	3.93 (1.99–7.79)	<0.001	1.66 (0.75–3.66)	0.210
**BM involvement vs. none**	3.08 (1.53–6.19)	0.002	1.04 (0.51–2.11)	0.910
**Splenomegaly vs. other**	5.21 (2.56–10.62)	<0.001	1.68 (0.77–3.66)	0.190
**Pleura effusion vs. other**	2.63 (0.8–8.69)	0.110		
**LDH elevation vs. none**	6.35 (3.18–12.71)	<0.001	2.08 (0.93–4.65)	0.073
**Hemoglobin <12 g/dL vs. ≥ 12 g/dL**	7.89 (3.99–15.59)	<0.001	2.3 (1.01–5.24)	0.048
**Ann Arbor Stage III–IV vs. I–II**	8.67 (2.64–28.48)	<0.001	2.82 (0.68–11.72)	0.150
**COVID-19 vs. none**	0.16 (0.02–1.17)	0.072		

BM, bone marrow; CI, confidence interval; HR, hazard ratio; HT, aggressive histologic transformation; LDH, lactate dehydrogenase; MCL, mantle cell lymphoma; MZL, marginal zone lymphoma; SM, secondary malignancy.

When considering competing risks, NLM was significantly associated with SMs, age over 60 years, male sex, pleural effusion, and elevated LDH level. The CIP analysis revealed that age over 60 years, MCL subtype, nodal MZL, and anemia were linked to poor outcomes related to lymphoma progression (Table 2).

### Detailed association analysis of risk factors

**Fig 2:**
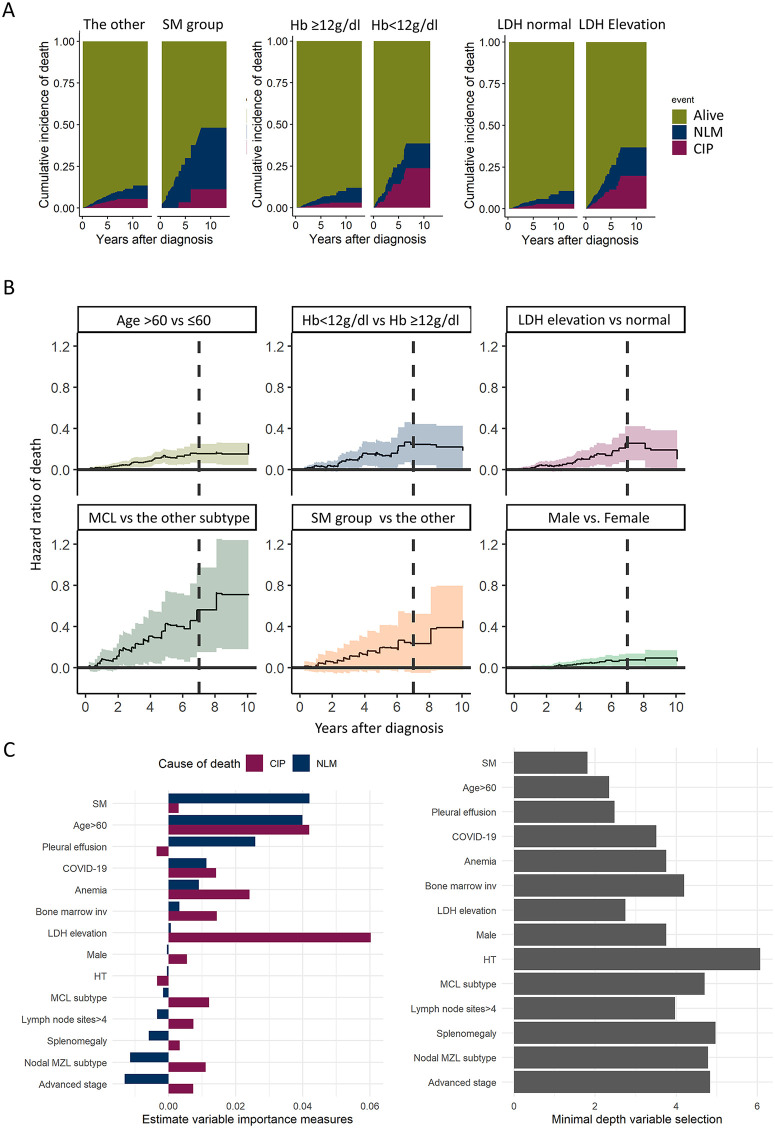
Effect of prognostic variables on survival outcomes. **(a)** Cumulative incidence of death by non-lymphoma-related mortality (NLM) and cumulative incidence of lymphoma progression death (CIP). **(b)** Aalen’s additive regression model demonstrated hazard ratios of mortality over time (years). The shaded region indicates the 95% confidence interval. **(c)** The bar plot shows estimated variable importance measures and minimal depth variable selection using a random forest survival model.

We compared NLM and CIP variables over time. In the SM group, NLM was more prevalent than CIP; the incidence rates of NLM at 2, 4, and 8 years were 10%, 18.9%, and 26.7%, respectively, whereas those of CIP were 0%, 3.4%, and 11.2%, respectively. However, CIP contributed more to total mortality than did NLM in the anemia group. The incidence rates of NLM at 2, 4, and 8 years were 5.1%, 9.9%, and 14.9%, respectively, whereas those of CIP were 3.5%, 14.2%, and 23.7%, respectively. An elevated LDH level showed similar associations with CIP and NLM (Fig 2A).

We applied Alan’s additive regression model to compare the effect of the factors by time course [21]. Covariates that consistently affected mortality were SM (average slope 0.06, p = 0.037), age over 60 years (slope 0.05, p < 0.001), male sex (slope 0.02, p = 0.028), and MCL subtype (slope 0.14, p = 0.002). These factors displayed a gradual positive slope, indicating a long-term effect on survival. For anemia (slope 0.07, p = 0.006) and an elevated LDH level (slope 0.05, p = 0.004), the slope of the plot showed the largest effect on mortality at approximately 7 years, with a decreasing effect thereafter (Fig 2B). A random forest survival model [22] was employed to further explore the time-varying nature of the model. The c-indexes for NLM and CIP were 0.79 and 0.77, respectively. The model identified the following key variables contributing to NLM: age over 60 years, SM, pleural effusion, and COVID-19. Similarly, the most important variables contributing to CIP were LDH level elevation, age over 60 years, anemia, nodal MZL subtype, and MCL subtype (Fig 2C).

These results highlight the complex interplay of factors influencing mortality in patients with LGBCLs and underscore the importance of considering lymphoma-related and non-lymphoma-related risk factors in prognostic assessments. Time‑varying Aalen overlays were used to refine the timing of these effects: SM contributed mainly mid‑term (peak at ~3.9 years, with attenuation thereafter); markers of burden/frailty (e.g., pleural effusion and age) peaked early and flattened, and anemia/LDH displayed smaller late‑tail signals (Fig S3 in S1 File).

### Survival analysis of risk factors by subtype

We compared risk factors by subtype, which is summarized in Table 3. In FL, elevated LDH independently predicted worse OS and non‑lymphoma mortality (NLM) (HR 3.65 and 2.52, respectively) as well as higher lymphoma‑progression mortality (CIP) (HR 6.46). Hb < 12 g/dL was associated with inferior OS and CIP, and pleural effusion showed an additional adverse signal. COVID‑19 showed a borderline association with NLM, while rituximab maintenance was protective for CIP (HR 0.21).

**Table 3:** Multivariate analyses of survival outcomes by subtype.

Follicular lymphoma						
	Overall survival		Non-lymphoma-related mortality		Cumulative incidence of lymphoma progression mortality	
	HR	P	HR	P	HR	P
**Age > 60 years vs. ≤ 60 years**			2.53 (0.99–6.42)	0.051		
**Lymph node >4 sites vs. 4 sites**	1.63 (0.81–3.30)	0.2			2.65 (0.75–9.41)	0.13
**Pleural effusion vs. other**			3.6 (1.05–12.28)	0.041		
**LDH elevation vs. none**	3.65 (1.77–7.52)	<0.001	2.52 (1.07–5.93)	0.034	6.46 (1.25–33.51)	0.026
**Hemoglobin <12 g/dL vs. ≥ 12 g/dL**	3.76 (1.83–7.71)	<0.001	1.79 (0.7–4.58)	0.22	5.35 (1.24–23.07)	0.025
**COVID-19 vs. none**			2.52 (0.94–6.78)	0.068		
**Rituximab maintenance vs none**					0.21 (0.06–0.77)	0.019
**MZL**						
**SM group vs. other**	6.37 (1.73–23.5)	0.005	10.74 (2.61–44.13)	0.001		
**HT group vs. other**	1.07 (0.23–4.97)	>0.999			3.04 (0.7–13.16)	0.14
**Age > 60 years vs. ≤ 60 years**	6.88 (2.41–19.7)	<0.001	10.5 (2.54–43.4)	0.0012	2.08 (0.16–26.67)	0.57
**Male vs. female**	2.27 (0.85–6.02)	0.1				
**Lymph node >4 sites vs. 4 sites**	4.45 (1.17–16.9)	0.029	4.59 (0.89–23.78)	0.069		
**BM involvement vs. none**	0.95 (0.37–2.46)	>0.999			2.07 (0.46–9.29)	0.34
**Splenomegaly vs. other**					0.8 (0.07–9.17)	0.86
**Pleural effusion vs. other**	5.2 (1.29–20.9)	0.02	15.75 (3.62–68.6)	<0.001		
**LDH elevation vs. none**			3.14 (0.93–10.6)	0.066	1.62 (0.36–7.25)	0.53
**Hemoglobin <12 g/dL vs. ≥ 12 g/dL**					1.58 (0.17–14.31)	0.69
**Ann Arbor Stage III–IV vs. I–II**					2.24 (0.33–15.05)	0.41
**Nodal MZL vs. other subtypes**	3.2 (1.25–8.19)	0.015				
**MCL**						
**SM group vs. other**			12.44 (3.61–42.86)	<0.001		
**HT group vs. other**						
**Age > 60 years vs. ≤ 60 years**	3.25 (0.89–11.9)	0.074			5.22 (0.79–34.6)	0.087
**Hemoglobin <12 g/dL vs. ≥ 12 g/dL**	3.32 (0.99–11.1)	0.051			1.57 (0.42–5.88)	0.51
**Blastoid subtype vs classic**	4.85 (1.54–15.3)	0.007			6.89 (1.8–26.35)	0.005

BM, bone marrow; CI, confidence interval; HR, hazard ratio; HT, aggressive histologic transformation; LDH, lactate dehydrogenase; MCL, mantle cell lymphoma; MZL, marginal zone lymphoma; SM, secondary malignancy.

**Fig 3:**
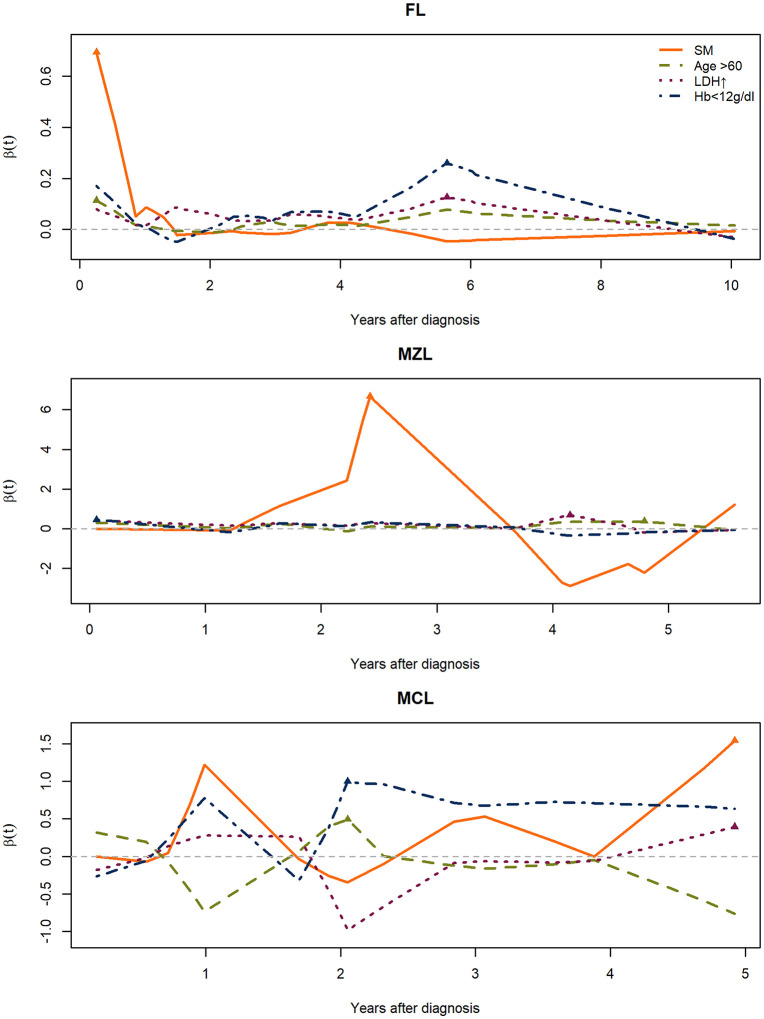
Time‑varying additive‑hazards effects by subtype. Panels show follicular lymphoma (FL), marginal zone lymphoma (MZL), and mantle cell lymphoma (MCL). Lines depict smoothed β(t) for second malignancy (SM), older age, lactate dehydrogenase (LDH) elevation, and anemia.

In MZL, second malignancy (SM) and pleural effusion were the strongest correlates—SM with both OS and NLM (HR 6.37 and 10.74) and pleural effusion with OS and NLM (HR 5.20 and 15.75)—with age > 60 years also strongly associated with OS/NLM; nodal MZL subtype and >4 nodal sites were related to OS. In the MCL subgroup, the treatment strategy followed the BRIGHT study, and most patients received BR- or R-CHOP–based chemo-immunotherapy. Autologous stem-cell transplantation was performed in five patients (12%) at the clinician’s discretion. Blastoid/pleomorphic morphology was observed in seven patients (15.2%) and was associated with poorer outcomes (OS HR 4.85; NLM HR 6.89). Second malignancy (SM) remained the strongest predictor of non-lymphoma mortality (HR 12.44) and showed a mid-term β(t) peak on Aalen analysis (Fig 3).

These findings suggest that adverse biology and SM mainly drive mortality risk in MCL. Time‑varying Aalen overlays showed an early short‑lived SM rise in FL, a mid‑term SM surge with volatility in MZL, and higher‑amplitude, short‑horizon signals for anemia/SM in MCL.

## Discussion

In this comprehensive analysis of 1,047 patients with LGBCLs, we identified key factors contributing to NLM and explored their time-varying effects. Our findings highlight the significant effect of SMs and emerging infectious risks, including COVID-19, on long-term outcomes in patients with LGBCLs [9,16].

The BRIGHT study, performed in North and South America and Australasia, prospectively followed treated patients with indolent non-Hodgkin lymphoma or MCL to assess long-term outcomes and reported 72 deaths: 31 from disease progression and 41 from NLM, consistent with the findings of our research [24]. The high incidence of NLM indicates the need to analyze the cause of death. The inclusion criteria of our study were based on those of the BRIGHT study. In addition, we included patients without chemotherapy to compare the impact of SMs, HT, and COVID-19.

Among the factors identified as important contributors to survival outcomes in the present study, older age—a factor included in the FIPI, MIPI, and MALT-IPI scores—mainly contributes to NLM and gradually worsens survival outcomes over time. An elevated LDH level is an essential contributor to NLM and CIP and is associated with inferior FLIPI, MIPI, and MALT-IPI scores. Anemia, which is only included in the FLIPI predictor, contributes to CIP, whereas the presence of SMs independently influences NLM. Among cancer types, hepatobiliary and colorectal cancers are the primary drivers of poor outcomes. Deaths in the SM group resulted from infection and SM-related complications. This finding emphasizes the importance of early detection of SMs and management of infection risks during treatment.

We acknowledge that HT is part of the natural progression of LGBCL rather than a separate disease. Nevertheless, we sought to determine whether the transformation itself has an independent prognostic effect and a distinct temporal pattern compared with non-transformed progression. Our univariate analysis indicated poor outcomes in the HT group; however, the multivariate analysis did not reveal that HT affected survival. This discrepancy could be due to overlapping effects and interactions between variables. For instance, factors such as an elevated LDH level and older age—factors included in other prognostic scores—may mask the independent effect of HT on survival outcomes. This finding aligns with that of the previous research by Wagner-Johnston et al., who identified LDH level elevation, advanced-stage B symptoms, and older age as negative prognostic features for HT arising from FL [25]. Furthermore, HT from MZL is associated with poor survival factors, such as LDH level elevation, male sex, low-performance status, nodal subtype, and older age [26].

In total, 20.6% of patients in our study were diagnosed with COVID-19. A subgroup analysis of patients who were followed-up or survived until 2021 showed that COVID-19 resulted in poorer survival outcomes in infected patients than in non-infected patients. Deaths from COVID-19-related pneumonia were most frequent in 2022, decreasing in 2023. In addition, the patients who received chemotherapy were more exposed to COVID-19-related infection. Further follow-up studies are needed to assess the long-term risks associated with COVID-19 in this patient population. We acknowledge that our COVID-19 analysis lacked detailed information on the vaccination status or timing. Because vaccine administration in Korea is managed centrally by the Korea Disease Control and Prevention Agency, such data were not uniformly retrievable across centers. However, from February 2021 onwards, vaccination was recommended for all oncology patients; almost all individuals receiving active therapy were presumed to have been vaccinated under this policy. Therefore, the minimal β(t) effect of COVID-19 observed in our analysis likely reflects the post-vaccination environment rather than a true absence of risk. This limitation has been noted, and further studies with explicit vaccination data are needed to delineate temporal vaccine effects more accurately.

Our findings have important clinical implications. Patients with FL, MZL, and MCL shared SMs and HT. HT is associated with lymphoma progression-related factors; in contrast, SM was a significant prognostic factor independent of other factors, except for age. Higher rates of SM have been reported in patients with non-Hodgkin lymphoma. Rituximab-induced B-cell depletion and decreased T-cell activity have been suggested to be responsible for increasing the prevalence of SMs [27]. Moreover, this immunocompromised status could make it challenging to treat SMs. The significant contribution of SMs and infectious complications to NLM suggests that management strategies for LGBCLs should extend beyond lymphoma control. The time‑varying analyses clarified phase‑specific risk. HT exerted its largest absolute impact early (~2 years), supporting front‑loaded surveillance and rapid escalation when transformation is suspected. SM contributed mainly in the mid‑survivorship window (~3–5 years) and was thereafter attenuated, reinforcing the need for structured second‑cancer screening/mitigation beginning by year 3. In contrast, the COVID‑19 curve was low‑amplitude and near zero, consistent with limited incremental absolute hazard in this cohort amid vaccination and improved therapeutics. Because these subgroups were small and heterogeneous and because risk sets diminish late in follow‑up, our findings are intended to provide guidance regarding when heightened vigilance is most warranted rather than to assert precise effect magnitudes.

In FL, early hazards clustered around frailty/tumor‑burden features (pleural effusion, LDH), with SM exerting only a brief effect—supporting front‑loaded supportive care and vigilance in the first 1–2 years, while recognizing the benefit of rituximab maintenance on CIP. In MZL, the concurrence of strong SM implies that mid‑term survivorship should prioritize second‑cancer screening/mitigation and cardiopulmonary/infection management. In MCL, outcomes were driven by biology (blastoid) and SM, consistent with more aggressive disease biology and a shorter effective horizon, advocating for early escalation and proactive management of comorbidity/secondary cancer risk.

The careful treatment of secondary primary malignancies in patients with LGBCLs requires personalized strategies, such as the selection of lesser-toxicity drugs or early initiation of antibiotic therapy [28]. Secondary-malignancy and non-lymphoma mortality should be attributed to multifactorial causes, including the cumulative exposure to alkylating or immunosuppressive agents (e.g., bendamustine), host vulnerability, and ageing. In line with current chemo-sparing treatment trends, therapy in indolent and mantle-cell lymphomas increasingly seeks to minimize cytotoxic exposure while maintaining durable control. Especially, chemo-less regimens in FL, such as rituximab plus lenalidomide, have shown durable long-term outcomes; furthermore, zanubrutinib-based chemo-less combinations in MCL are emerging as promising therapeutic strategies [29,30]. However, the potential for increased infection-related death might affect long-term survival. Future studies should consider the risk of immune suppression. Furthermore, humoral immune reconstitution, such as intravenous immunoglobulin infusion, could be a complementary approach [31].

Our study has some limitations. First, the follow-up time for confirming the effect of SMs on OS was relatively short; in particular, the impact of SMs with a long latency, such as breast or bladder cancer, could not be determined [14]. However, we presented the survival outcome of each subtype and highlighted the targets of caution. Second, we excluded patients with chronic lymphocytic leukemia, lymphoplasmacytic lymphoma, and Waldenstrom macroglobulinemia because these diseases could bias the study outcomes. Exclusion criteria were established because the initiation of treatment for chronic lymphocytic leukemia (for example, treatments involving Bruton’s Tyrosine Kinase inhibitors or B-cell lymphoma-2 inhibitors) differs from that of other LGBCLs [32]. Another reason for the exclusion of these diseases is that, unlike other LGBCLs, they have distinct prognostic features, such as the level of immunoglobulin M or the mutational status of the immunoglobulin heavy chain gene [33,34]. Third, some patients with MCL exhibit distinct inferior outcomes, characterized by blastic and pleomorphic variants, a high Ki-67 index, and *TP53* mutations [35]. Given the lack of these specific data in our study, we conducted a multivariate analysis of MCL subtypes of blastoid/pleomorphic variants to account for this confounding factor and address this limitation. In the MCL subgroup, all patients received rituximab-based chemo-immunotherapy, mainly BR or R-CHOP, following the treatment framework of the BRIGHT study, which evaluated these regimens across indolent B-cell lymphomas including MCL. This homogeneous therapeutic background allowed the assessment of clinical and biological factors affecting mortality under a comparable treatment intensity. However, we acknowledge that this approach does not reflect the full spectrum of contemporary MCL management. Fourth, the relatively young median age in our cohort compared with that in Western series probably reflects the younger population structure of Korean lymphoma registries. In addition, early diagnosis through broad insurance coverage and routine health screening may have led to the inclusion of clinically healthier patients. This trend could partly underestimate age-associated mortality and limit direct comparability with older international cohorts; therefore, age effects in our results should be interpreted cautiously and validated in broader, population-based settings. Fifth, because this was a retrospective, incompletely characterized cohort, outputs from our advanced statistical models (competing‑risk and time‑varying Aalen analyses, and any machine‑learning summaries) were used to describe patterns and generate hypotheses rather than to draw definitive causal conclusions. We therefore restricted interpretation to the timing and direction of effects, treated small subgroups and late‑follow‑up fluctuations with caution, and viewed external validation in prospectively profiled cohorts as essential. Finally, genomic classification approaches that affect survival were not analyzed [36–38]. However, we focused on clinical features because the main focus was on effects beyond lymphoma progression. Considering the retrospective nature of this study, further prospective trials are needed to confirm its findings.

## Conclusion

This study provides a nuanced perspective on mortality risk factors in patients with LGBCLs, emphasizing the critical role of non-lymphoma-related causes of death. Our findings underscore the dynamic nature of these risks over time, challenging the traditional static approach to LGBCL prognostication. Previously reported prognostic models have typically incorporated factors associated with the early stages of long-term survival, with aggressive histologic changes often linked to survival outcomes. By integrating SMs, infectious complications, and established prognostic factors into a comprehensive risk assessment model, we aimed to identify more enduring factors that can impact patient care throughout the patient’s lifetime. This approach paves the way for personalized, time-adaptive management strategies for patients with LGBCLs. Moving forward, the focus should shift toward developing tailored surveillance protocols and targeted interventions that address lymphoma progression and non-lymphoma-related risks. Such a paradigm shift in LGBCL management can substantially enhance long-term outcomes, improving the survival and quality of life of patients with this chronic malignancy.

## Supporting information

S1 File Supplementary Figures S1–S4. (DOCX)
