# Supplementary material for: Comprehensive analysis of mortality risk factors in low-grade B-cell lymphoma
Source: PLoS One. 2026 Mar 4;21(3):e0328666. doi: 10.1371/journal.pone.0328666 (PMC12959707; doi:10.1371/journal.pone.0328666)
Supplement: S1 File — (DOCX) [file pone.0328666.s001.docx]

**Supporting Information**

Comprehensive analysis of mortality risk factors in low-grade B-cell lymphoma

Tong-Yoon Kim ^1^, Gi-June Min ^2^, Seok-Goo Cho ^2^, Seoree Kim ^3^, Jong Hyuk Lee ^4^, Byung-Su Kim ^5^, Joon Won Jeoung ^6^, Hye Sung Won ^7^, and Youngwoo Jeon ^1^*

^1^ Department Of Hematology, Yeoido St. Mary’s Hospital, College of Medicine, The Catholic University of Korea, Seoul, Republic of Korea

^2^ Department Of Hematology, Seoul St. Mary’s Hospital, College of Medicine, The Catholic University of Korea, Seoul, Republic of Korea

^3^ Department Of Oncology, Bucheon St. Mary’s Hospital, College of Medicine, The Catholic University of Korea, Seoul, Republic of Korea

^4^ Department Of Hematology, Incheon St. Mary’s Hospital, College of Medicine, The Catholic University of Korea, Seoul, Republic of Korea

^5^ Department Of Hematology, Eunpyeong St. Mary's Hospital, College of Medicine, The Catholic University of Korea, Seoul, Republic of Korea

^6^ Department Of Oncology, Daejeon St. Mary’s Hospital, College of Medicine, The Catholic University of Korea, Seoul, Republic of Korea

^7^ Department Of Oncology, Uijeongbu St. Mary’s Hospital, College of Medicine, The Catholic University of Korea, Seoul, Republic of Korea

***** Correspondence: native47@catholic.ac.kr; Tel.: +82(2) 3779-1039 (Y.-W.J.)

**S1 Fig.** CONSORT flow diagram.


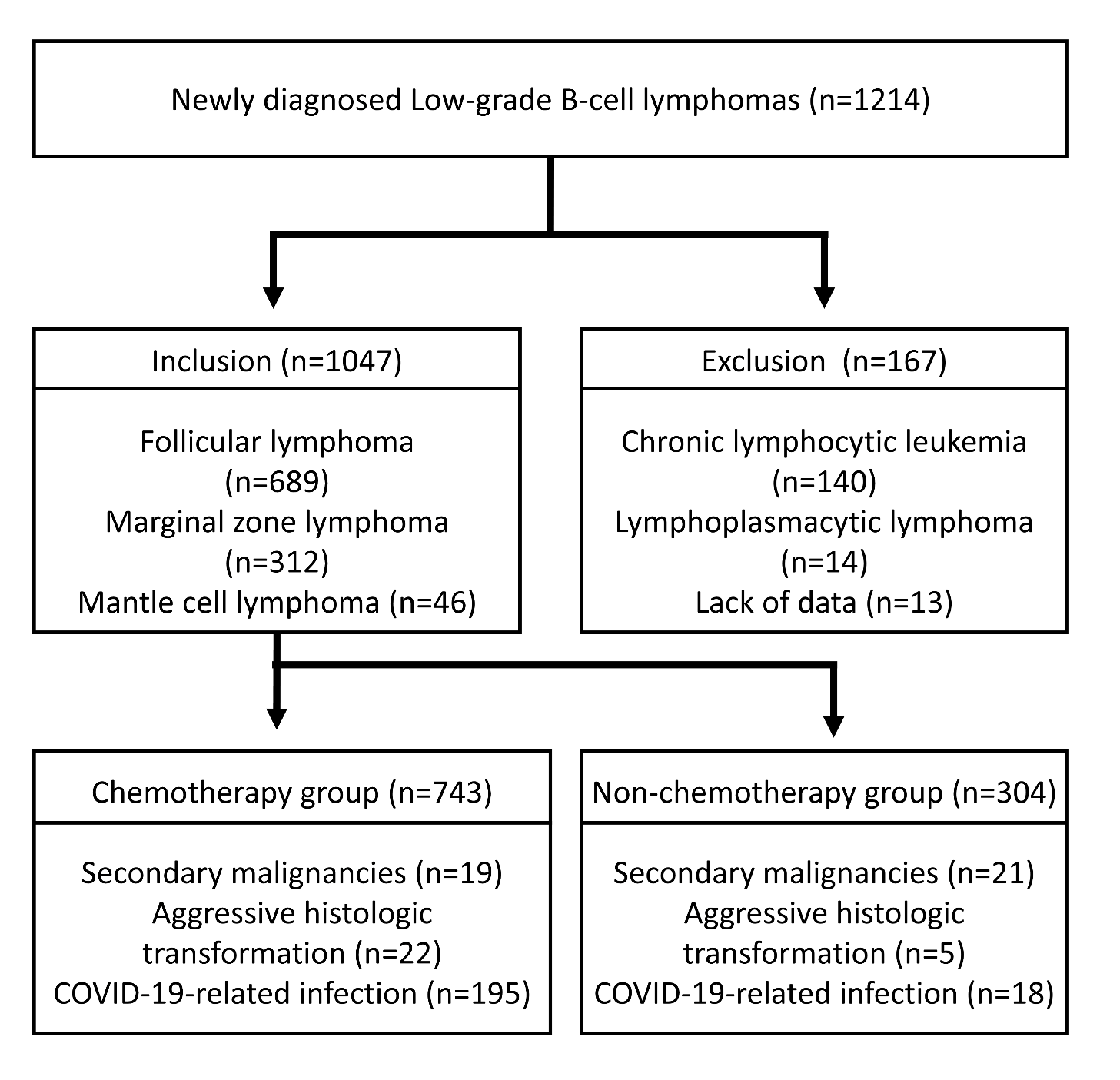


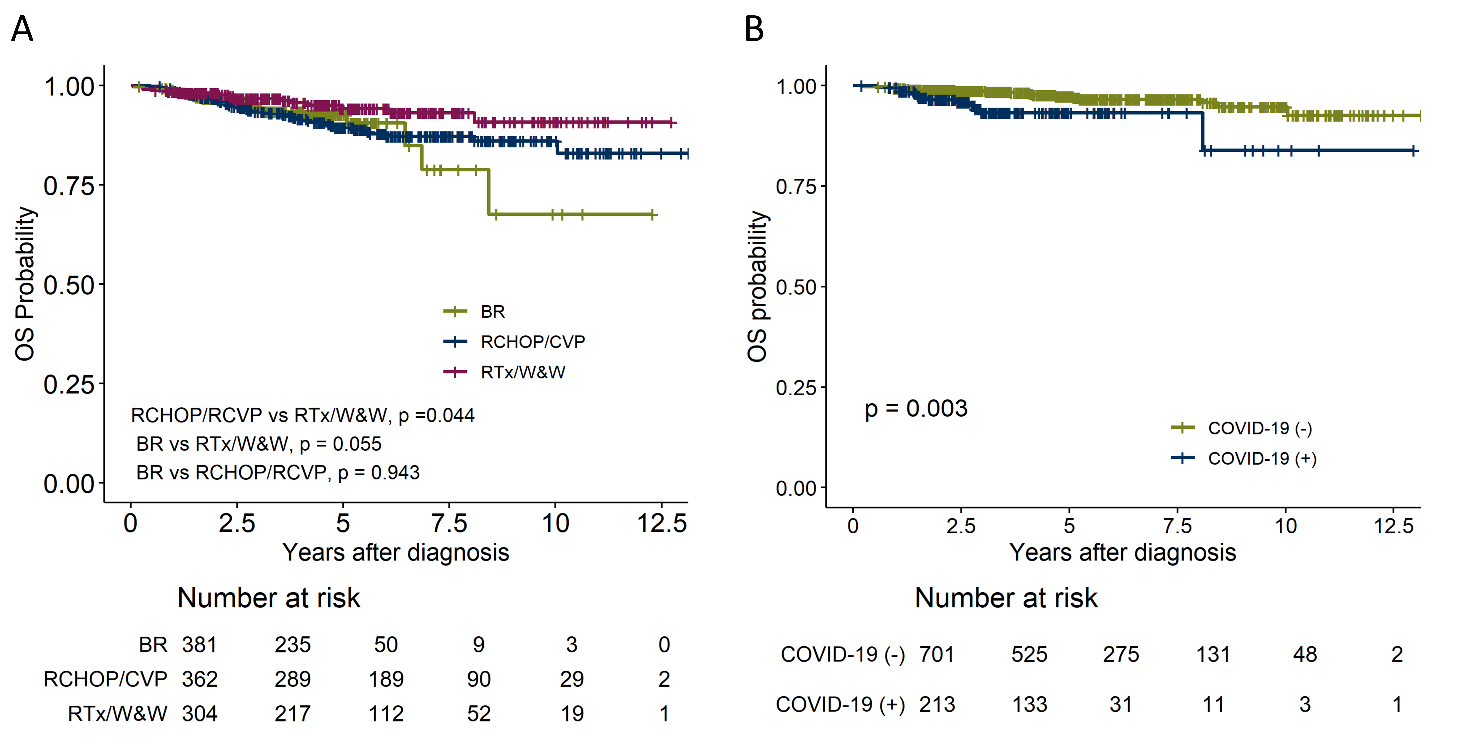


**S2 Fig.** Survival outcomes of patients with low-grade B-cell lymphoma (LGBCL). (A) Overall survival (OS) by the first-line treatment, (B) OS by coronavirus disease 2019 (COVID-19) infection. BR, bendamustine and rituximab; RCVP, rituximab, cyclophosphamide, vincristine, and prednisolone; RTx, radiotherapy; W&W, watch and wait

**
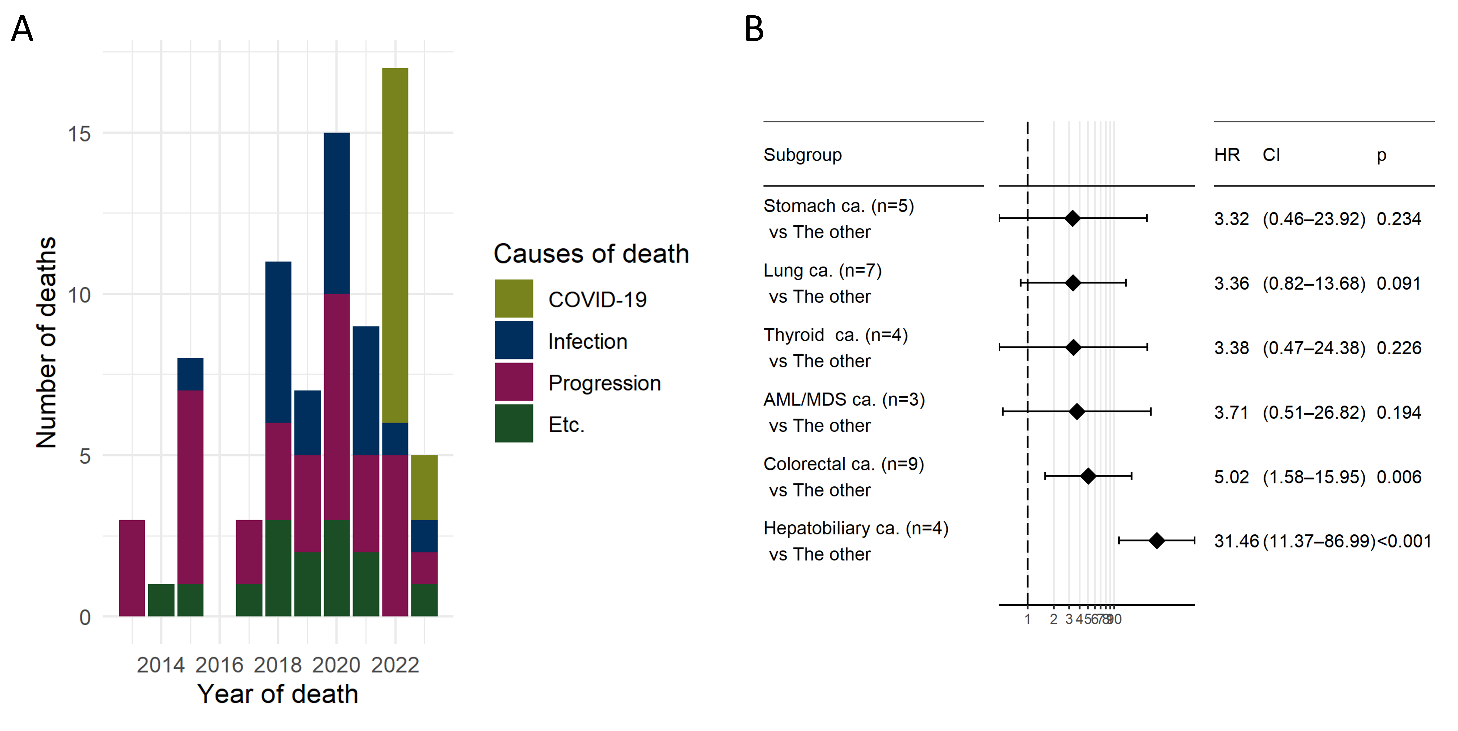
**

**S3 Fig.** Causes of death in patients with low-grade B-cell lymphoma. (A) Bar plot of causes of mortality by the year. (B) Forest plot of HR by secondary malignancy subtype. HR, hazard ratio; CI, confidence interval

**
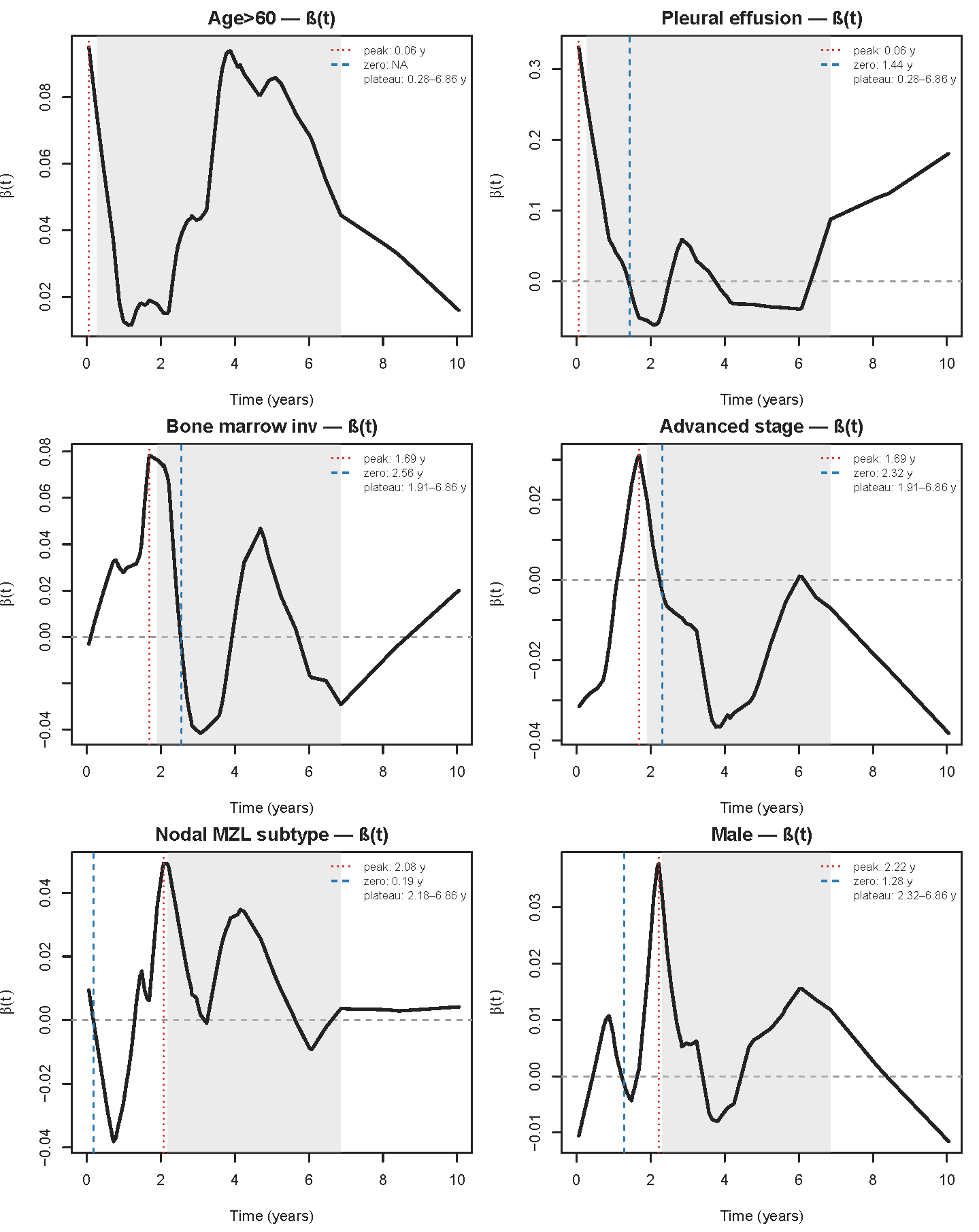
**

**
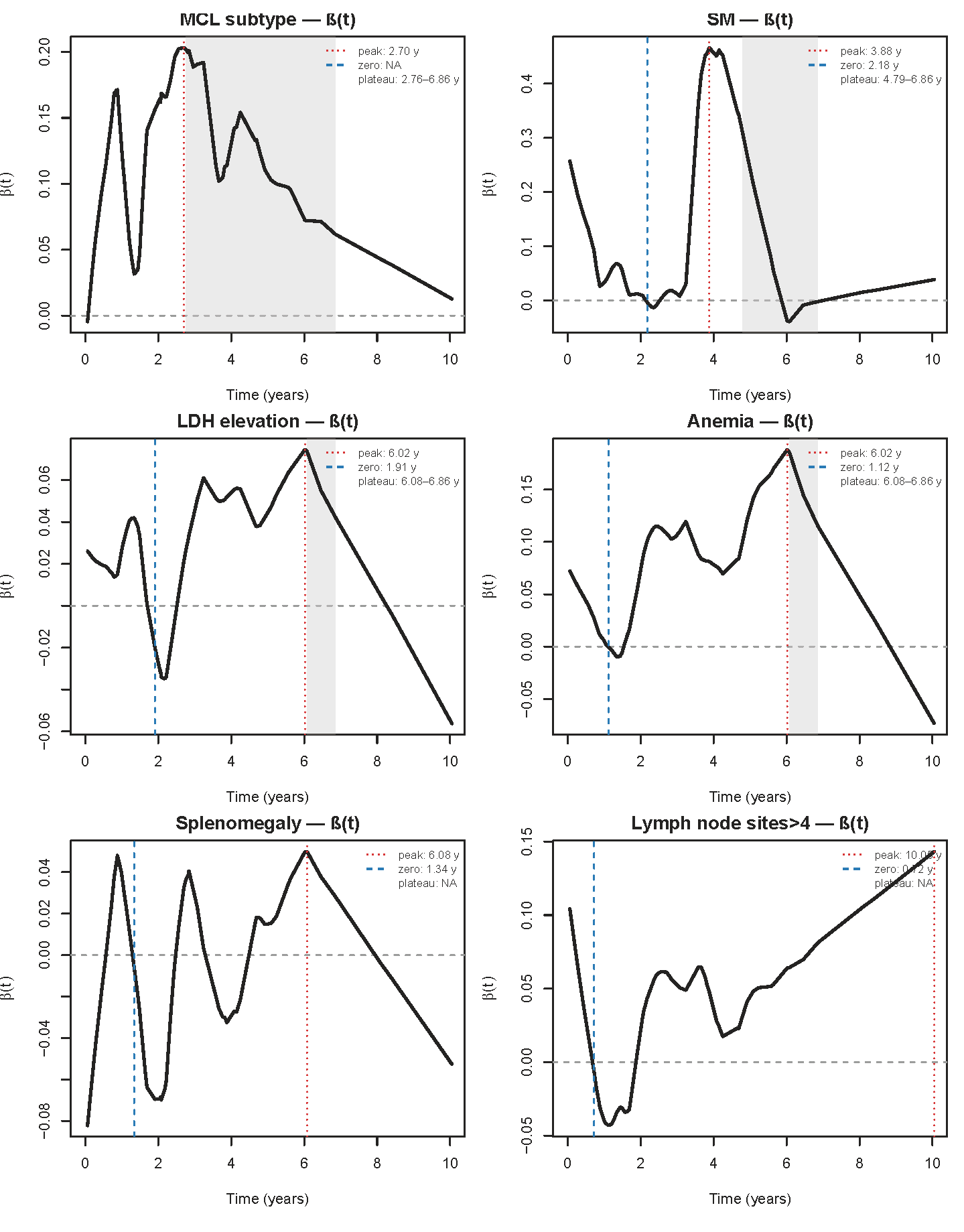
**

**S4 Fig.** Time-varying additive-hazards effects for clinical and biological covariates.

Red dashed line marks the time of maximum positive effect (t_peak), blue dashed line indicates the first zero-crossing after a positive value, and the grey shaded area represents the plateau interval—the longest post-peak segment where the centered 7-point moving average of |β(t)| remained below a data-adaptive threshold (ε_frac = 0.03; α = 0.01). The y-axis represents β(t), the instantaneous absolute risk difference at time t.
